# Adult Hemolytic Uremic Syndrome as a Diagnostic Challenge

**DOI:** 10.7759/cureus.87406

**Published:** 2025-07-07

**Authors:** Adarsh A Madharia, Sagar Khadanga, Seema P Mahant, M. Sukumar

**Affiliations:** 1 General Medicine, All India Institute of Medical Sciences, Bhopal, Bhopal, IND

**Keywords:** acute kidney injury, adult hus, hemolytic uremic syndrome, microangiopathic hemolytic anemia, thrombocytopenia

## Abstract

Hemolytic uremic syndrome (HUS) is a rare but serious condition in adults, typically characterized by a triad of microangiopathic hemolytic anemia (MAHA), thrombocytopenia, and acute kidney injury (AKI). While most commonly associated with pediatric populations and Shiga toxin-producing *Escherichia coli* (STEC), adult-onset cases remain underrecognized, especially in resource-limited settings like India.

We report the case of a 35-year-old male patient who presented with a low-grade fever, bloody diarrhea, jaundice, and dark brown-colored urine. Laboratory evaluation revealed MAHA (hemoglobin: 9.8 g/dL, schistocytes >2%, elevated lactate dehydrogenase), and thrombocytopenia (platelet count 45,400/mm³), and acute kidney injury (AKI) (serum creatinine: 0.45 mg/dL initially, with transient rise). Notably, direct bilirubin was disproportionately elevated (6.5 mg/dL), raising suspicion of hepatic involvement. Blood cultures grew *Escherichia coli* (*E. coli)*, though stool cultures were negative. Peripheral smear, elevated reticulocyte count, and absence of disseminated intravascular coagulation (DIC) supported the diagnosis of typical HUS. ADAMTS13 levels, complement assays, and stool Shiga toxin testing were unavailable. The patient also had severe vitamin B12 deficiency, raising consideration of pseudo-thrombotic microangiopathy (pseudo-TMA), which was concurrently treated. He later developed non-cardiogenic pulmonary edema, managed with corticosteroids and supportive therapy. He was discharged on hematinics, and follow-up at two months revealed hemoglobin improvement to 10.2 g/dL and full renal recovery. This case highlights the diagnostic and therapeutic challenges in adult HUS, especially with overlapping features of pseudo-TMA and potential infectious triggers. The absence of definitive diagnostic modalities like ADAMTS13 testing necessitated reliance on clinical scoring, peripheral smear, and exclusion of mimickers. The elevated direct bilirubin and rare pulmonary involvement broadened the clinical complexity. Early recognition and supportive care led to favorable outcomes. Yet the inability to completely exclude atypical causes such as *Streptococcus pneumoniae*-associated HUS (Sp-HUS) and B12 deficiency imposes a clinical challenge. Adult HUS remains a diagnostic challenge due to its rarity, atypical presentations, and limited access to confirmatory testing in many settings. This case emphasizes the need for systematic evaluation, awareness of differential diagnoses like pseudo-TMA and Sp-HUS, and timely supportive management. It also contributes to the growing literature on adult HUS in India, underscoring the importance of clinical vigilance in atypical hemolytic syndromes.

## Introduction

Hemolytic uremic syndrome (HUS) is a clinical condition characterized by the triad of microangiopathic hemolytic anemia (MAHA), thrombocytopenia, and acute kidney injury (AKI) [1]. It is considered a subset of thrombotic microangiopathies (TMA), a heterogeneous group of disorders unified by this triad but differing in etiology, pathogenesis, and outcomes [2].

HUS encompasses multiple etiologies, including Shiga-toxin-associated HUS (STEC-HUS or typical HUS), atypical HUS (aHUS) due to complement dysregulation, *Streptococcus pneumoniae*-associated HUS (Sp-HUS), and rare metabolism-mediated forms, such as those due to cobalamin deficiency or other inborn errors [3,4]. While STEC-HUS is classically associated with children, recent outbreaks have demonstrated that adults, too, can be significantly affected. A notable example is the 2011 German outbreak caused by *Escherichia coli *(*E. coli*) O104:H4, which predominantly affected adults [5].

In Europe, the annual incidence of HUS in adults is estimated to be 0.5-one per 100,000 population; incidence may increase in adults during outbreaks caused by *E. coli* [3]. In India, the incidence of HUS has primarily been documented in pediatric populations, with limited literature on adult cases. Adult HUS is often underrecognized and may be misdiagnosed, delaying life-saving interventions. This case report highlights the clinical presentation, diagnostic challenges in differentiating it from mimics such as vitamin B12 deficiency and disseminated intravascular coagulation (DIC), and management of HUS in an adult patient in central India. By contributing to the sparse literature on adult HUS in this setting, we aim to emphasize the importance of early recognition and tailored management strategies.

## Case presentation

We describe a case of a 35-year-old male patient who presented with a 20-day history of low-grade, undocumented fever associated with chills and a non-productive cough. Two days before admission, he developed blood-stained loose stools, followed by yellowish discoloration of the eyes and dark brown-colored urine a day prior to hospital presentation.

He was initially evaluated at a private hospital, where investigations revealed bicytopenia and hyperbilirubinemia (hemoglobin: 6.0 g/dL; platelet count: 50,000/μL; total bilirubin: 8.12 mg/dL; indirect bilirubin: 1.67 mg/dL; direct bilirubin: 6.45 mg/dL). The elevated direct (conjugated) bilirubin was suggestive of hepatic dysfunction or cholestasis rather than hemolysis per se. He also had rising serum creatinine (4.35 mg/dL), indicating AKI, and received two units of packed red blood cells. He was then referred to our tertiary care center in Central India for further management.

On admission, he had pallor and icterus. Vitals revealed a pulse rate of 124 beats/minute and blood pressure of 136/84 mmHg. Systemic examination was notable only for diffuse mild abdominal tenderness; other findings were unremarkable.

Empirical therapy with intravenous doxycycline and cautious fluid management was initiated. Persistent hemoglobin decline led to evaluation for hemolysis. Peripheral blood smear showed 2.8% schistocytes, lactate dehydrogenase (LDH) was elevated at 1292 U/L, and indirect bilirubin was 9.09 mg/dL. The reticulocyte count was elevated at 21.32%, consistent with intravascular hemolysis, although haptoglobin levels were not available. However, markedly elevated direct bilirubin also pointed to concurrent liver dysfunction.

Coombs testing was deferred due to prior transfusions, which could yield false positives. The coagulation profile, including prothrombin time/international normalized ratio (PT/INR), activated partial thromboplastin time (aPTT), and fibrinogen, was normal, ruling out DIC. Trends of laboratory investigations are summarized in Table 1.

**Table 1:** Trends of hematological and biochemical investigations during the hospital stay SGOT: Serum glutamic-oxaloacetic transaminase; SGPT: serum glutamic pyruvic transaminase

Parameter	Reference range	Unit	Day 1	Day 2	Day 3	Day 4	Day 6	Day 10	Day 11
Hemoglobin (Hb)	12–16	g/dL	8.0	7.9	7.1	6.9	5.8	5.4	6.5
Total leukocyte count (TLC)	4–10	×10⁹/L	4.0	6.08	8.75	6.75	9.31	16.02	17.80
Differential leukocyte count (DLC)	Neutrophils 40–75, Lymphocytes 20–45, Monocytes <10	%	89/7/3	90/6/3	87/9/2	-	-	76/13/10	-
Platelets (PLT)	150–400	×10⁹/L	17	29	39	47	26	107	169
Hematocrit (HCT)	36–46	%	27.8	25.2	23.2	23.2	-	-	-
Schistocytes (SCX)	<1%	%	-	-	2.8	-	0.5	-	-
Urea	15–45	mg/dL	96.6	134.78	137.24	118.50	68.7	35.6	-
Serum creatinine (S. Cr)	0.6–1.3	mg/dL	4.26	3.46	2.82	2.18	-	0.72	-
Sodium (Na)	135–145	mmol/L	133	140	135	136	136	136	-
Potassium (K)	3.5–5.0	mmol/L	4.49	3.68	4.21	4.32	4.06	4.45	-
Chloride (Cl)	98–106	mmol/L	103.5	103	102	103	104	100	-
Total bilirubin (TB)	0.3–1.2	mg/dL	-	20.77	19.15	17.29	16.19	6.33	-
Direct bilirubin (DB)	0–0.3	mg/dL	-	11.68	11.04	10.26	10.88	3.20	-
Aspartate aminotransferase (AST, SGOT)	<40	IU/L	-	116.8	67.2	69.5	53.3	57.1	-
Alanine aminotransferase (ALT, SGPT)	<40	IU/L	-	69.9	48.1	39.5	30.0	31.7	-
Alkaline phosphatase (ALP)	44–147	IU/L	-	207.2	170.4	136.5	-	176.8	-
Total protein (T.PR)	6.4–8.3	g/dL	-	5.04	4.93	4.78	4.35	5.52	-
Albumin (ALB)	3.5–5.0	g/dL	-	2.91	2.75	2.58	2.16	2.27	-
Lactate dehydrogenase (LDH)	140–280	IU/L	-	1292	1101	-	-	461.9	-

Extensive evaluation to identify the etiology of TMA was undertaken. Stool cultures for *Vibrio*, *Shigella*, and *E. coli* were negative; however, blood cultures grew *E. coli*, raising suspicion for STEC-HUS. The patient had no history of drug intake (e.g., chemotherapeutics, immunosuppressants) to suggest drug-induced HUS.

Further workup revealed a severe vitamin B12 deficiency. Since B12 deficiency can cause pseudo-TMA with schistocytosis, anemia, and thrombocytopenia, this was also considered. However, the clinical and biochemical profile, along with *E. coli* bacteremia, supported a diagnosis of STEC-HUS with concurrent B12 deficiency. He was started on vitamin B12 supplementation.

Chikungunya IgM was positive. While the Chikungunya virus is not typically associated with hemolysis, rare cases of TMA-like presentation have been reported; hence, it was considered an incidental finding. Given a fever duration of more than five days, NS1 antigen testing for dengue was not relevant; IgM enzyme-linked immunosorbent assay (ELISA) would have been preferable if dengue was suspected. Detailed serological, biochemical, and microbiological investigations are summarized in Table 2.

**Table 2:** Additional serological, biochemical, and microbiological investigations RBC: red blood cell; WBC: white blood cell; MPICT: malaria parasite immunochromatographic test; ELISA: enzyme-linked immunosorbent assay; IFA: indirect immunofluorescence assay

Day	Investigation	Result	Reference range/ Interpretation
Day 1	Peripheral blood smear	Few fragmented red cells, 3–4 nucleated RBCs/100 WBCs; Corrected reticulocyte count: 21.32%	Schistocytes <1%; Retic: 0.5%–2.5%
Day 2	Peripheral blood smear	Few fragmented red cells, 2–3 nucleated RBCs/100 WBCs	Schistocytes <1%
Day 2	Vitamin B12	95 mg/dL	200–900 mg/dL
Day 2	Vitamin B9 (Folate)	15 mg/dL	>4 mg/dL
Day 2	Urine culture and sensitivity	Sterile	No growth
Day 3	Malaria (MPICT)	Negative	Negative
Day 3	Leptospira IgM ELISA	Equivocal	Negative
Day 3	Scrub typhus IgM ELISA	Negative	Negative
Day 3	Serum iron	117.97 µg/dL	60–170 µg/dL
Day 3	Total iron-binding capacity (TIBC)	159.14 µg/dL	240–450 µg/dL
Day 3	Transferrin saturation	74.13%	20–50%
Day 3	Ferritin	>2000 ng/mL	30–400 ng/mL
Day 4	Chikungunya IgM ELISA	Positive	Negative
Day 4	Dengue NS1 ELISA	Negative	Negative
Day 4	Stool culture	No *Salmonella*, *Shigella*, or *Vibrio* spp. grown	No pathogen growth
Day 6	Hepatitis A IgM ELISA	Negative	Negative
Day 6	Hepatitis E IgM ELISA	Negative	Negative
Day 6	Hepatitis D IgM ELISA	Negative	Negative
Day 6	Antinuclear antibody (ANA) by IFA	Negative	Negative

**Figure 1:**
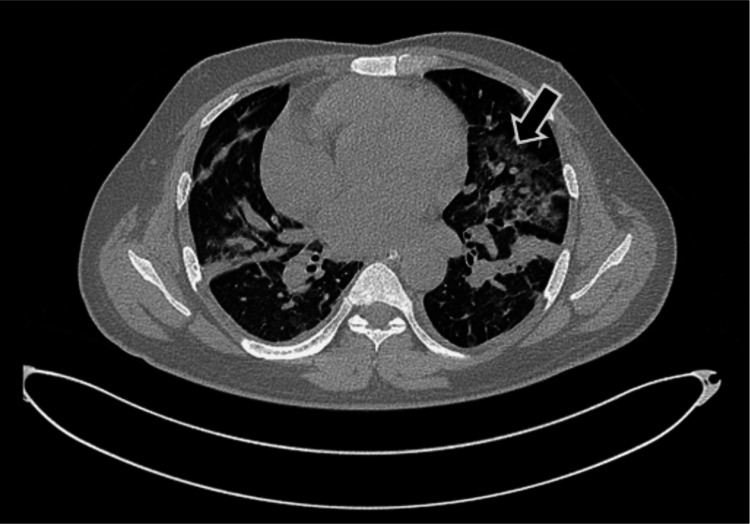
High-resolution computed tomography (HRCT) of the chest (axial view) showing bilateral patchy areas of ground-glass opacities (arrow). These findings are suggestive of non-cardiogenic pulmonary edema, a rare complication of hemolytic uremic syndrome (HUS), although some changes may also reflect pre-existing chronic lung pathology.

On day 6, the patient developed worsening respiratory distress requiring noninvasive ventilation. High-resolution computed tomography (HRCT) of the chest (Figure 1) revealed multiple patchy ground-glass opacities and pleuroparenchymal bands in both lungs. These changes, although suggestive of non-cardiogenic pulmonary edema, an unusual complication of HUS, also raised the possibility of underlying chronic lung disease. A pre-discharge or follow-up HRCT could not be obtained.

Meropenem was initiated based on culture sensitivity. Oseltamivir and oral corticosteroids were started for respiratory worsening. The patient showed gradual clinical and laboratory improvement, with recovery of renal function and hemoglobin.

He was discharged on oral hematinics and vitamin B12 supplementation. On follow-up after two months, his hemoglobin had improved to 10.2 g/dL, with stable renal function and no new symptoms.

## Discussion

HUS is a toxin-mediated MAHA that, while predominantly reported in pediatric populations, remains relatively rare in adults. It falls under the broader category of TMAs, a heterogeneous group of disorders defined by the clinical triad of MAHA, thrombocytopenia, and end-organ damage [1]. HUS includes STEC-HUS, complement-mediated aHUS, Sp-HUS, and metabolism-related pseudo-TMAs such as those caused by vitamin B12 deficiency [2,4].

The pathophysiology of STEC-HUS involves ingestion of Shiga toxin-producing *E. coli* (STEC), which penetrates the intestinal mucosa and binds to Gb3 receptors in endothelial cells, particularly in the glomeruli. This leads to endothelial injury, cytokine release, complement activation, and ultimately microangiopathy and thrombosis [3]. The most frequently implicated serotypes are O157:H7 and O104:H4, the latter responsible for a major European outbreak in 2011 that affected a large number of adults [5].

Our adult patient, presenting with short-duration bloody diarrhea, anemia, thrombocytopenia, AKI, and positive blood cultures for *E. coli*, met the diagnostic criteria for HUS. Though stool cultures were negative for STEC, the absence of detectable Shiga toxin is not uncommon by the time HUS manifests [6]. This highlights a practical challenge in resource-limited settings like India, where definitive testing (e.g., ADAMTS13 activity, Shiga toxin assays, and complement levels) is often unavailable. In our case, we followed the Indian Society of Pediatric Nephrology (ISPN) recommendations for clinical diagnosis: hemoglobin <10 g/dL, schistocytes >2%, elevated LDH, thrombocytopenia, and rising creatinine, all of which were present [7].

A notable finding in our patient was significantly elevated direct bilirubin (6.5 mg/dL), suggestive of conjugated hyperbilirubinemia, typically associated with cholestasis or hepatic dysfunction rather than intravascular hemolysis, which more commonly causes indirect hyperbilirubinemia. This atypical presentation warranted careful differential consideration. Additionally, although intravascular hemolysis may lead to hemoglobinuria, it does not always result in overt hyperbilirubinemia, and bilirubin patterns can vary [8].

The presence of severe vitamin B12 deficiency, a known cause of pseudo-TMA, added further complexity. B12 deficiency can cause ineffective erythropoiesis, red cell fragmentation, schistocytosis, and thrombocytopenia [9]. Although not the primary etiology in this case, pseudo-TMA should always be considered and addressed appropriately. Our patient received hematinics upon discharge and demonstrated improvement in hemoglobin to 10.2 g/dL at a two-month follow-up.

Non-cardiogenic pulmonary edema, observed in our patient and confirmed by HRCT, though rare, is a known complication of HUS. It may arise from oligoanuria, volume overload, or severe hypertension [10]. In our case, respiratory symptoms responded to steroids and supportive care. Sp-HUS, another possible etiology of pulmonary involvement, was not identified as blood cultures grew *E. coli* and not *Streptococcus* *pneumoniae* [11].

Due to the unavailability of ADAMTS13 assays, risk scores like PLASMIC or French scores, which help distinguish thrombotic thrombocytopenic purpura (TTP), were not applied. These scoring systems are particularly useful in resource-constrained environments and should be considered when laboratory facilities are limited [12]. Direct Coombs test (DCT) and indirect Coombs test (ICT) were not performed as the patient had received packed red cell transfusions, which may result in false positives, although recent evidence by Froissart et al. has suggested that blood investigations, including the Coombs test, are still useful in determining the cause of anemia after one blood transfusion [13].

While complement levels are crucial in identifying atypical HUS and guiding eculizumab use, they were not tested as the clinical picture was consistent with typical HUS. Despite controversy over the role of antibiotics in STEC-HUS due to the risk of increased toxin release, our patient improved after treatment with appropriate antimicrobials, likely because the indication was bloodstream infection with *E. coli* [14].

HRCT findings also included pleuroparenchymal bands and atelectasis, suggestive of possible pre-existing lung disease. A pre-discharge HRCT could have clarified these chronic changes, but was not performed.

Literature on adult-onset HUS is limited, especially in Indian settings. Most reports focus on pediatric or Western cohorts. Kouzy et al. and Wong et al. documented longer prodromal diarrheal phases, whereas our patient had a short course, highlighting presentation variability [15,16]. Outcomes are generally favorable in typical HUS, provided timely diagnosis and supportive care.

## Conclusions

This case illustrates the diagnostic and therapeutic complexities of adult-onset HUS in a resource-limited setting. The overlapping features of pseudo-TMA due to vitamin B12 deficiency, the unusual presentation of pulmonary edema and direct bilirubinemia, and the absence of confirmed STEC in stool cultures, along with the unavailability of ADAMTS13 and haptoglobin, all underscore the challenges clinicians face in real-world scenarios. Nonetheless, adherence to systematic diagnostic algorithms, vigilance for complications, and prompt supportive management, including the use of antibiotics, facilitated a positive outcome. This report adds valuable insight to the limited body of evidence on adult HUS in India and underscores the need for greater awareness and diagnostic access to modalities such as ADAMTS13 assays in order to improve outcomes in such patients.
